# Stoichiometric and stable isotope ratios of wild lizards in an urban landscape vary with reproduction, physiology, space and time

**DOI:** 10.1093/conphys/coaa001

**Published:** 2020-02-14

**Authors:** Andrew M Durso, Geoffrey D Smith, Spencer B Hudson, Susannah S French

**Affiliations:** 1 Department of Biology and the Ecology Center, Utah State University, 5305 Old Main Hill, Logan UT 84321 USA; 2 Department of Biological Sciences, Florida Gulf Coast University, 10501 FGCU Blvd S, Fort Myers, FL 33965 USA; 3 Biological Sciences Department, Dixie State University, 225 S. University Avenue, St. George, UT 84770 USA

**Keywords:** body condition, carbon, community ecology, corticosterone, ecosystem ecology, fasting, immunity, nitrogen, nutritional stress, oxidative stress, urbanization, *Uta stansburiana*

## Abstract

Spatial and temporal variation in stoichiometric and stable isotope ratios of animals contains ecological information that we are just beginning to understand. In both field and lab studies, stoichiometric or isotopic ratios are related to physiological mechanisms underlying nutrition or stress. Conservation and ecosystem ecology may be informed by isotopic data that can be rapidly and non-lethally collected from wild animals, especially where human activity leaves an isotopic signature (e.g. via introduction of chemical fertilizers, ornamental or other non-native plants or organic detritus). We examined spatial and temporal variation in stoichiometric and stable isotope ratios of the toes of *Uta stansburiana* (side-blotched lizards) living in urban and rural areas in and around St. George, Utah. We found substantial spatial and temporal variation as well as context-dependent co-variation with reproductive physiological parameters, although certain key predictions such as the relationship between δ^15^N and body condition were not supported. We suggest that landscape change through urbanization can have profound effects on wild animal physiology and that stoichiometric and stable isotope ratios can provide unique insights into the mechanisms underlying these processes.

## Teaser

The chemical signatures of the bodies of lizards contain information about what they eat. We compared lizard chemical signatures between urban and rural areas in southern Utah and found that they have much broader diets in urban areas and that some chemical signatures were related to reproduction.

## Introduction

Landscape change through urbanization is a major factor influencing the distribution, conservation status, foraging behaviour and nutritional status of wildlife (Ditchkoff *et al.*, 2006). Our understanding of some of the mechanisms driving the negative effects of urbanization on wildlife is becoming more complete (e.g. Bradley and Altizer, 2007, McKinney, 2008). Nutritional stress due to habitat loss or alteration can contribute to wildlife declines (Suorsa *et al.*, 2004, Amo *et al.*, 2006, Naug, 2009, Vangestel *et al.*, 2010). However, most studies of how nutrition affects survival take place away from urban centres, and few studies of urban wildlife address nutrition.

Nutritional stress is a common challenge faced by wild animals. In some wildlife populations, nutritional stress can compromise individual health and thereby decrease survivorship, with potential conservation implications (McNamara and Houston, 1990, Wikelski and Romero, 2003, Romero and Wikelski, 2010). Starvation is a major source of mortality for some wildlife (Stout and Cornwell, 1976, Young, 1994, Krebs *et al.*, 2004), particularly when populations are subject to unpredictable temporal variation in resource availability (Romero and Wikelski, 2001, Monteith *et al.*, 2014) or only have access to resources of poor quality (Knapp *et al.*, 2013, Murray *et al.*, 2015). Warmer winters may increase energetic demand during hibernation, eating into energy budgets (Zani *et al.*, 2012).

Measuring nutritional stress in wild animals can be challenging. Biologists desire rapid, non-invasive measurement techniques for assessing nutritional stress in wild animals (Romero and Reed, 2005, Romero and Reed, 2008, Speakman, 2008). Although several such techniques are available (reviewed in McCue, 2010), including measuring stable isotope and stoichiometric composition, lipid profiles, hormones and circulating metabolites, consensus about their meaning has not been reached (Sarre *et al.*, 1994, Dickens and Romero, 2013). Few studies have measured more than one of these endpoints simultaneously, although proximate haematological measurements underlying energy allocation among physiological demands might be expected to co-vary with isotope and stoichiometric ratios, both of which are ultimately underpinned by variation in nutritional stress. Reptiles represent a useful model for ecosystem ecology. Recently, studies of reptiles in urban areas have proliferated (Audsley *et al.*, 2006, Mitchell *et al.*, 2008, French *et al.*, 2018). These ectothermic vertebrates have relatively limited capacity for dispersal but often persist in urban areas due to their remarkable plasticity in response to environmental change (e.g. Glanville and Seebacher, 2006, Refsnider and Janzen, 2012, Ackley *et al.*, 2015). Although the role of wildlife as sentinels in environmental change is becoming more widely recognized (Hopkins, 2007), studies of the urban ecology of reptiles still lag behind those of other vertebrates. Variable resource availability can have major effects on reptile populations (Romero and Wikelski, 2001), although their poikilothermic metabolism can allow individuals to persist through relatively long periods of resource scarcity (Willson *et al*., 2006).

Both stoichiometric ratios and stable isotope ratios integrate ecological information over relatively long periods of time compared with other metrics. They can offer unique insights into the ecology of amphibians and reptiles (Willson *et al.*, 2010), which can be very cryptic, often have low detection probabilities (Durso and Seigel, 2015, Rodda *et al.*, 2015), and whose metabolic flexibility may limit the utility of plasma metabolites for predicting recent feeding history (Price, 2017). Changes to elemental and isotopic ratios appear to result from mobilization, reorganization and catabolism of stored lipid and protein reserves during fasting, especially decreases in tissue lipid concentrations during the later stages of fasting (McCue, 2010). Empirical data support relationships between carbon-to-nitrogen ratio (C:N) and body condition (Graves *et al.*, 2012) or environmental stressors (Zhang *et al.*, 2016) and between stable carbon (^13^C:^12^C or δ^13^C) and nitrogen (^15^N, ^14^N or δ^15^N) isotope ratios and body condition, nutritional stress (Hatch, 2012, Mekota *et al.*, 2006) or environmental factors (Hartman, 2011). Controlled laboratory studies of reptiles report that nutritional stress causes isotopic enrichment (McCue and Pollock, 2008, Martinez del Rio *et al.*, 2009), but the utility of C:N and stable isotope ratios for monitoring the nutritional status of wild reptiles is still largely untested, and the relationships between these metrics and other physiological endpoints are unknown.

Here, we explored relationships among stoichiometric and isotopic ratios and physiological and morphological parameters of wild lizards living across an urban landscape. Although several studies have found relationships between stable isotope ratios of reptiles and aspects of their ecology (Barrett *et al.*, 2005, Reddin *et al.*, 2016), none have yet to focus on changing landscape parameters or physiology of free-living vertebrates. We examined a large data set on the stoichiometric (C:N) and stable isotope (δ^13^C and δ^15^N) ratios of wild lizards from populations that vary in their exposure to urbanization and anthropogenic stressors. We compared these biochemical markers to other commonly used measures of physiological stress (immunocompetence, clutch size, glucocorticoid concentrations, oxidative stress) within each population and assessed the generality of these relationships. We hypothesized that lizards from each site would differ in their absolute isotopic composition, but that systematic variation between urban and rural sites would be present, and that isotope and stoichiometric ratios would be related to at least one physiological parameter in our large data set, at least at some sites or in some years.

## Methods

### Site description

We collected wild *Uta stansburiana* (side-blotched lizards) from six locations (three urban and three rural) in and around St. George, Utah, USA, every May for 5 years (*N* = 592; see Table 1 and map in Fig. S1 for more details). All sites are rocky areas ≤ 1.8 ha in size near or along riparian corridors near the intersection of the Great Basin, Colorado Plateau and Mojave Desert. The six sites lay an average of 21 km from one another (minimum = 4 km; maximum = 42 km; Fig. S1). The dominant vegetation surrounding the three rural sites consists of *Juniperus osteosperma* (Utah juniper) and *Atriplex confertifolia* (shadscale), with smaller amounts of *Pinus edulis* (pinyon pine), *Pinus monophylla* (single-leaf pinyon), *Artemisia* (sagebrush), *Amelanchier* (serviceberry), *Cercocarpus* (mountain mahogany), *Pinus ponderosa* (ponderosa pine) and *Purshia* (cliffrose) (Table 2). Surrounding the three urban sites, the dominant vegetation was once *Larrea tridentata* (creosotebush), with smaller amounts of *Ambrosia* (bursage), *Coleogyne* (blackbrush) and *Mahonia fremontii* (Fremont mahonia), but is now largely converted to city and cultivated land (Table 2). On average, the human population density is two orders of magnitude lower at rural sites, there are more than four times fewer kilometres of roads within a 2-km^2^ radius and the percentage of human-impacted watercourses is more than three times as high (Table 2). All three urban sites are along a heavily used cycling and walking path, and many feral cats are seen in the immediate vicinity (although larger lizards [*Crotaphytus*, *Gambelia*, *Sceloporus uniformis*], snakes, roadrunners, corvids, kestrels and mesomammals are present at all sites). We calculated a metric of disturbance, ‘total area of active human use’, by digitizing the total area covered by man-made urban structures, trails and roads within a 250-m buffer of each site from 2013, 2015 and 2017 Google Earth imagery; this metric was more than five times higher at urban sites than at rural sites (Table 2).

For the past several years, the St. George metropolitan area has been among the fastest growing in the USA and is currently ranked fifth in terms of percentage growth, with an increase in population of 24.3% from 2010 to 2018 (U.S. Census Bureau, 2019). One urban site (U2) was completely developed into a parking lot in late 2015; we nevertheless searched for lizards there in 2016 but found none (Table 1). In order to control for year-to-year variation in precipitation, which ultimately controls almost all aspects of the ecology of this very arid desert ecosystem, we also collected data on local rainfall in the 12 months preceding each of our sampling occasions from the National Oceanic and Atmospheric Administration (2018; Table 3).

**Table 1:** Sample sizes from all sites in all years

	Urban Sites			Rural Sites			Total
Year	U1	U2	U3	R1	R2	R3	
2013	20	30	8	18	20	19	115
2014	21	19	16	19	18	29	122
2015	12	15	19	13	22	13	94
2016	13	0^*^	29	19	11	22	94
2017	36	0^*^	36	36	30	29	167
Total	102	64	108	105	101	112	592

All sites are rocky areas ≤ 1.8 ha in size near or along riparian corridors. ^*^One urban site was completely developed into a parking lot in late 2015; we nevertheless searched for lizards there in 2016 but found none

**Table 2:** Comparison of anthropic characteristics of urban (*n* = 3) and rural (*n* = 3) sites

	Urban sites			Rural sites		
Measure of anthropogenic influence	U1	U2	U3	R1	R2	R3
Human population density (people/km^2^) in surrounding 2 km^2^ area	777.8	873.6	392.8	0.0	0.9	18.2
km of roads in surrounding 2 km^2^ area	102	103	80	9	28	28
% impacted watercourses in surrounding 2 km^2^ area	45%	54%	51%	0%	16%	32%
Total area of active human use (m^2^) within 250 m	189 595	56 169	101 119	8326	30 592	28 668
Elevation (m a.s.l.)	775	790	766	1304	1242	1179
Dominant vegetation	City (51%), cultivated land (39%), creosotebush (10%)	Cultivated land (86%), city (10%), creosotebush (5%)	Creosotebush (95%), city (5%)^*^	Utah juniper (100%)	Utah juniper (82%), shadscale (18%)	Utah juniper (55%), shadscale (45%)

A buffer of 2 km^2^ was chosen because this was the largest distance that prevented buffers from overlapping. U.S. Census data from 2010 were used to calculate population density. ^*^Dominant vegetation data are the most recent available, from 2001; since this time, the area around site U3 has been developed

**Table 3:** Rainfall in the 12 months preceding each of our sampling occasions (which took place in early May)

Year	2012	2013	2014	2015	2016	2017
Cumulative rainfall (mm)	143	201	197	98	298	290

We did not sample in 2012, but it is included because it may be relevant to sampling that took place in 2013. Data from National Oceanic and Atmospheric Administration (2018)

### Sample collection

We captured lizards using lightweight poles with a loop of fishing line or dental floss with a running knot attached to one end, which tightened once placed around the neck of a lizard. We collected one to four whole toes from each lizard, dried them to a constant mass (0.5–2.0 mg) using a drying oven set to 60 °C, and wrapped them in tin capsules (5 × 9 mm; Costech Analytical). All samples were stored in a drying oven for 1–2 months. Because each lizard had a different code for mark-recapture, a different combination of one to four toes was collected from every individual. We expect this variation to be random with respect to isotopic composition. Lizard toes and insects were not ground. Plants were ground using a Wig-L-Bug Dental Amalgamator. Samples were not lipid-extracted because we wanted to test whether the C:N of bulk tissues was a reliable indicator of body condition and nutritional stress, as shown in other taxa (Hayashi, 1983, Okumura *et al.*, 2002, Post *et al.*, 2007, Graves *et al.*, 2012). All samples were analyzed using continuous-flow direct-combustion mass spectrometry on an isotope-ratio mass spectrometer (Europa Scientific ANCA-2020; PDZ, Crewe, England) at the Utah State University Stable Isotope Lab. We measured δ^13^C and δ^15^N as well as the total mass of C and N, from which we calculated the C:N ratio. Standard reference materials (glucose ammonium sulphate) were used for calibration. Standard deviations of replicate standards (calculated as relative standard deviation of 5–10 standard precision tests) did not exceed 0.1 per mil. We removed seven outlying data points that might have been influenced by measurement error (δ^15^N > 20‰).

In addition to recording the sex of each lizard, we also recorded their colour morph (Sinervo and Lively, 1996), which has been shown to be polymorphic with respect to reproductive strategy, home range size and other aspects of ecology in some parts of the range of *U. stansburiana* (e.g. Comendant *et al.*, 2003, Mills *et al.*, 2008). We also measured their mass (to the nearest 0.1 g using a digital balance and a plastic cup), snout-vent length (to the nearest mm using a plastic ruler), and the clutch size and follicle dimensions (to the nearest 0.1 mm) of the females using an ultrasound (MicroMaxx, SonoSite, Bothell, WA, USA). Within 3 min of capture, we collected blood samples (plasma volume 1–81 mL, median = 28 mL, mean ± SD = 31 ± 13 mL) from the retro-orbital sinus using a glass capillary tube, from which we measured bacterial killing ability (BKA; French and Neuman-Lee, 2012), corticosterone (CORT; Neuman-Lee *et al.*, 2015) and two components of oxidative stress: reactive oxygen metabolites (dROM) and plasma oxidative barrier (OXY; Diacron International, Grosseto, Italy). In 2013, we collected a second blood sample 10 min after the first to measure the difference between baseline and post-stress corticosterone (CORT reactivity; Moore *et al.*, 1991, French *et al.*, 2008, Lucas and French, 2012). Because each toe clip was unique, recaptures of individually marked and released lizards allowed us to prevent pseudoreplication. We calculated a body condition index using the residuals of a regression of snout-vent length and mass.

Because geographic variation in the isotopic signatures of plants and invertebrates propagates to higher trophic levels (Pilgrim, 2005), we did not make comparisons of absolute values among sites, but rather analyzed within-site variation in isotopic signature as it related to other physiological endpoints of condition and stress. No site was larger than 1.8 ha in size, and we assumed that spatial variation in isotopic signature within a site was negligible. To test whether spatial variation in source was important among sites, we collected isotopic data on plants (*N* = 41 in 2014 and *N* = 108 in 2017) and whole insects (*N* = 9 in 2014 and *N* = 39 in 2017) collected at each site. We attempted to collect the same species of plants and insects across sites, but because the plant and insect communities varied so much from site to site, we were forced to use ecological equivalents in some cases (Table S1). At each site, we attempted to collect representatives of the dominant plant species present. We queried the TRY curated global database of plant traits (Kattge *et al.*, 2011) to assemble data on photosynthetic pathway, using consensus data from congeners when an exact species match was not available (Table S1). In 2014, our insect collection included just ants of the genus *Pogonomyrmex*, which are conspicuous and abundant members of the ant community at all sites, forage widely, thus accumulating their own food from many species of plants, and make up a large proportion of the diet of *U. stansburiana* in other ecosystems (Knowlton, 1934, Knowlton and Nye, 1946, Tinkle, 1967, Best and Gennaro, 1984), although many additional ant genera with differing ecologies are also members of local ant assemblages, and *U. stansburiana* feed on a wide variety of invertebrates other than ants. Although stable isotope variation within ant species and even within colonies has been documented (Tillberg *et al.*, 2006, Roeder and Kaspari, 2017), we did not examine such variation at our sites. In 2017, we expanded our insect collection to include other ants, other hymenopterans, orthopterans, dipterans, ephemeropterans and other insects (Table S1). Within-site variation in ant δ^13^C and δ^15^N from 2014 to 2017 was small (Fig. S2). We executed a simple two-source mixing model for each site using the package siar (v. 4.2; Parnell and Jackson, 2011) to examine the proportion of lizard toe tissue derived from C_3_ vs C_4_/CAM plants, using the site-specific mean and SD δ^13^C and δ^15^N values of C3 and C4/CAM plants as the two sources and TEF values of 6 ± 0.5 for N and 2 ± 0.05 for C (Post, 2002).

### Data analysis

Differences in non-isotope/non-stoichiometric physiological measurements between urban and rural sites were evaluated by Smith (2017) and are described briefly below. In order to evaluate co-variation between isotopic/stoichiometric ratios and other physiological measurements, we modelled three response variables (C:N, δ^13^C, δ^15^N) individually against 10 continuous explanatory variables (SVL, mass, body condition, reproductive investment [clutch mass; females only], BKA, CORT, dROM, OXY and the other two isotopic/stoichiometric ratios), with fixed block effects for sex (two levels, except in the reproductive investment model), year (five levels; Table 1) and site (six levels; Table 1). Although site and year could be considered random, we chose to model them as fixed because we wished to estimate effect sizes for each site and year. We also tested whether the same three response variables differed among colour morphs (three levels) or between lizards that were later recaptured (known survivors) and those that were not (two levels) using the ‘Anova’ function in the ‘car’ package (Fox and Weisberg, 2011). We used the package ‘nlme’ in R (Pinheiro *et al.*, 2016, R Core Team, 2019, version 3.2.2) for modelling and the package ‘ggplot2’ to make figures (Wickham, 2009).

## Results

**Figure 1:**
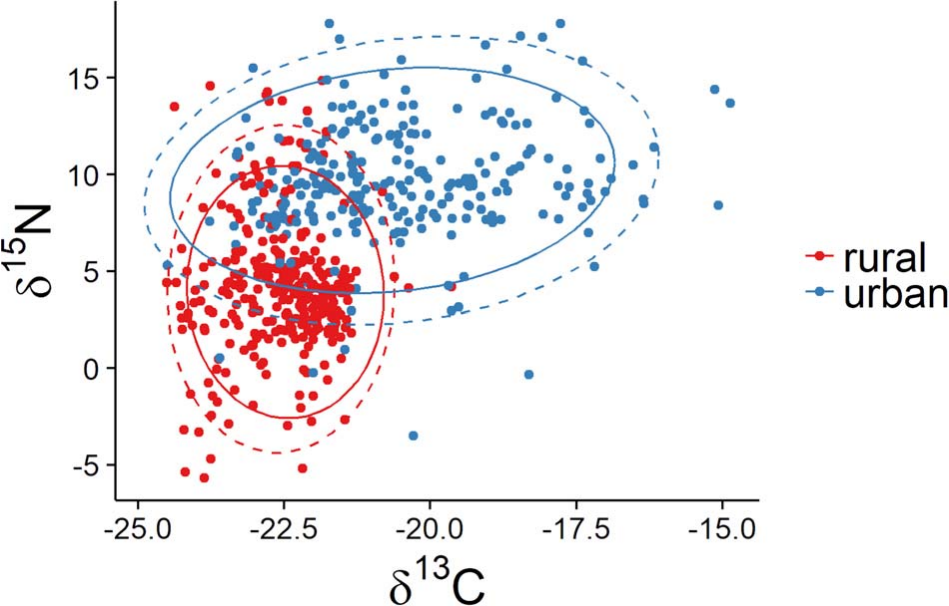
Stable carbon and nitrogen isotope ratios of toes of side-blotched lizards (*Uta stansburiana*) at three urban and three rural sites in southwestern Utah, with normal 95% confidence ellipses assuming a multivariate normal (solid) and multivariate *t*-distribution (dashed), computed using stat_ellipse in R

**Figure 2:**
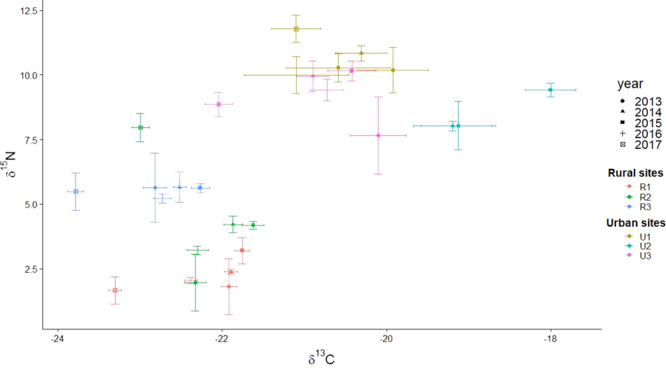
Annual variation in stable carbon and nitrogen isotope ratios of toes of side-blotched lizards (*Uta stansburiana*) at three urban and three rural sites in southwestern Utah

**Figure 3:**
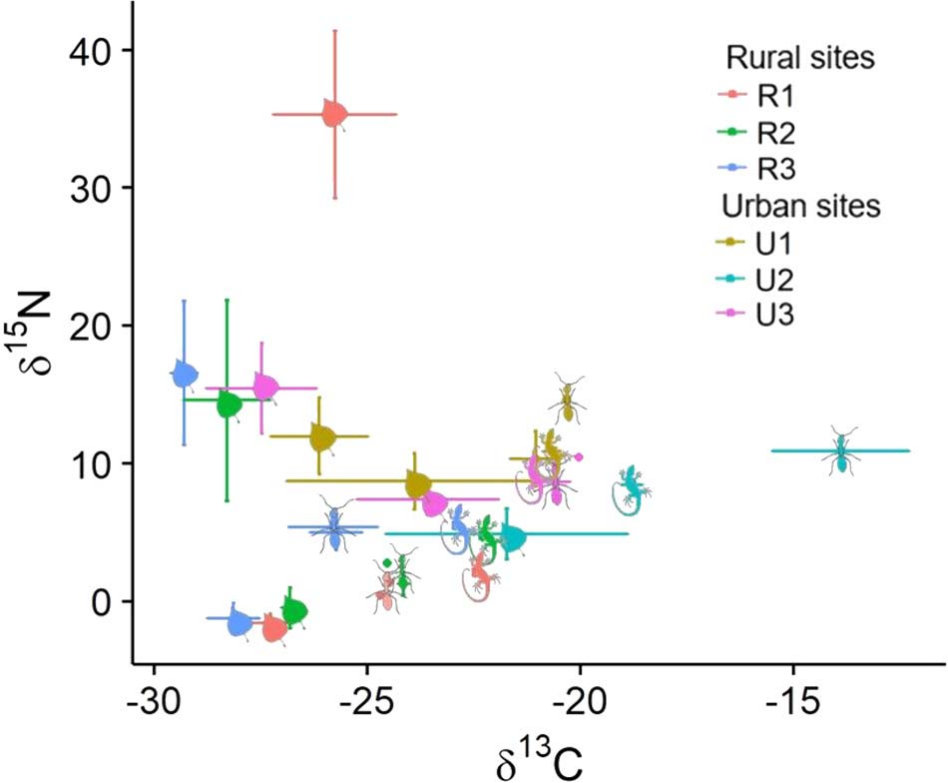
Close and consistent relationship between stable carbon and nitrogen isotope ratios of plants, ants, and toes of side-blotched lizards (*Uta stansburiana*) at three rural, but not three urban sites in southwestern Utah

**Figure 4:**
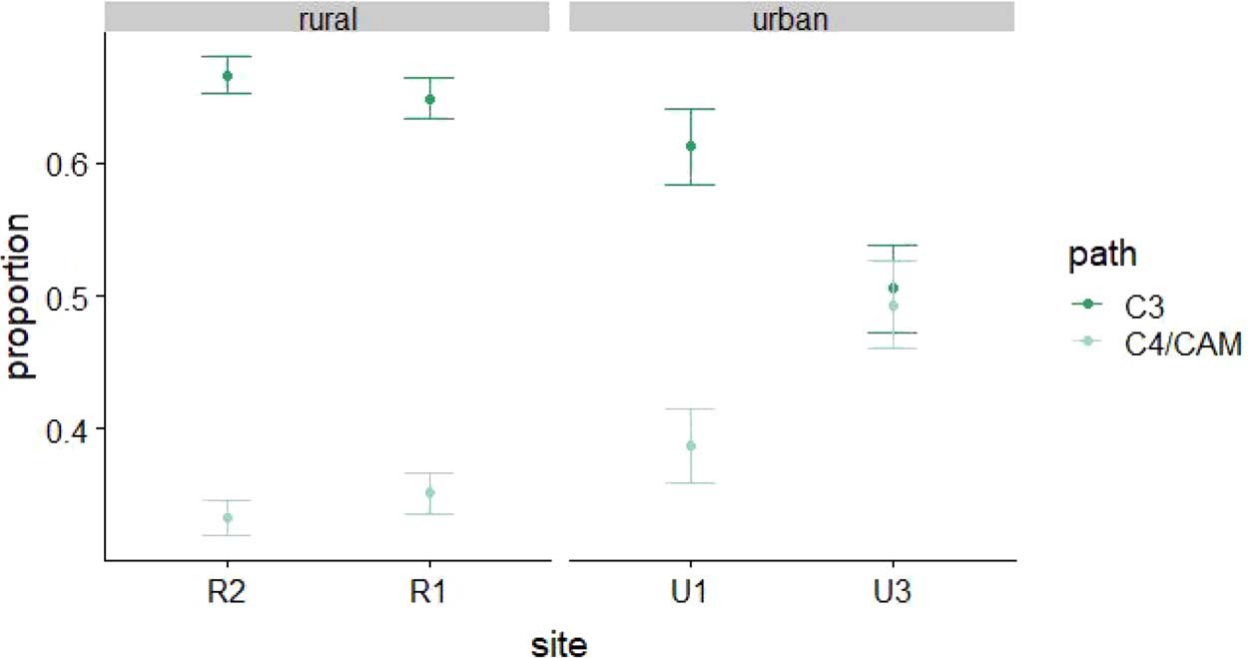
Proportion of side-blotched lizard (*Uta stansburiana*) toe tissue predicted to be derived from plants with C_3_ and C_4_/CAM photosynthetic pathways at two rural and two urban sites. Site-specific output from siarmcmcdirichletv4 in package siar (Parnell and Jackson, 2011) with 200 000 iterations and a burn-in of 50 000, thinning by 15. We used TEF values of 6 ± 0.5 for N and 2 ± 0.05 for C. Sites U2 and R3 are not shown because we collected two few C_4_/CAM plants to model

We found that lizards from different sites differed from one another in δ^13^C, δ^15^N, and C:N ratio (Table 4; Fig. 1). The variation in δ^13^C was much higher at urban sites than at rural sites (Fig. 1). Annual variation was present but lesser in magnitude than spatial variation and consistent across sites (Fig. 2). Stable isotope signatures of ants were closely and consistently paired with those of lizard toes at all three rural sites (Fig. 3). We observed differences between ants and lizard toes of δ^13^C = 2.6–3.3 and δ^15^N = 0.24–1.06 at rural sites, whereas at urban sites, we observed differences between ants and lizard toes of δ^13^C = −4.9–0.11 and δ^15^N = −4.08 to −1.20. Plant isotopic signatures at all sites were dominated by C_3_ species. The proportion of lizard tissue predicted to be derived from C_3_ vegetation was slightly higher at rural (65–66%) than at urban (52–60%) sites (Fig. 4). At three of the four sites with sufficient plant sampling, the posterior probability distribution for C_3_ plants did not overlap that of C_4_/CAM (Fig. 4).

**Table 4:** Type III ANOVA table showing variation among sites, years, and sexes

δ^13^C			δ^15^N			C:N		
Factor	*F*_df_	*P*	Factor	*F*_df_	*P*	Factor	*F*_df_	*P*
Site	114_5,539_	**<0.00001**	Site	118_5,539_	**<0.00001**	Site	6.56_5,539_	**<0.00001**
Year	34.1_4,539_	**<0.00001**	Year	5.3_4,539_	**<0.0005**	Year	14.39_4,539_	**<0.00001**
Sex	1.62_1,539_	0.200	Sex	1.18_1,539_	0.280	Sex	0.42_1,539_	0.520

Significant *P* values are highlighted in bold. Interactions were not significant

**Figure 5:**
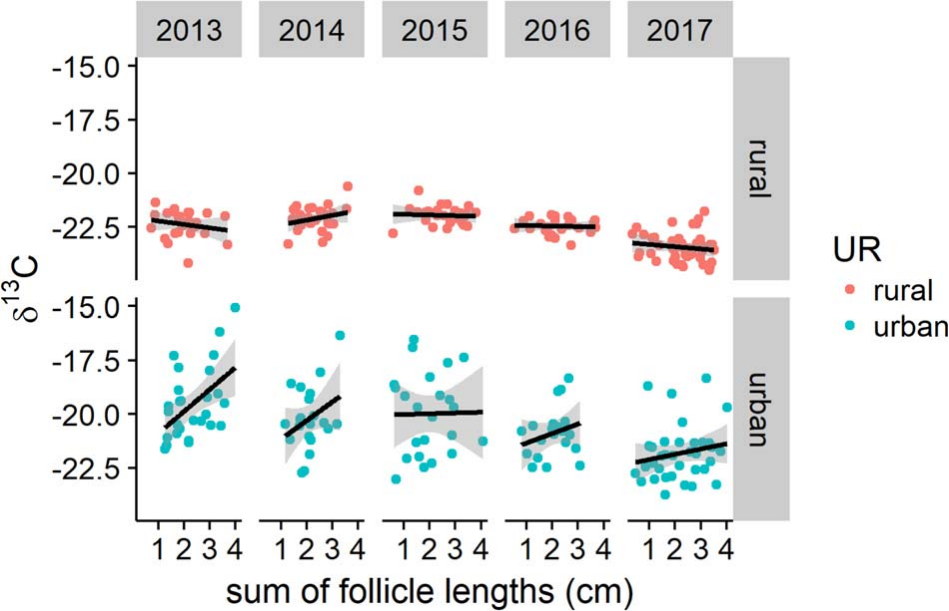
Co-variation of toe δ^13^C ratio and clutch size of female side-blotched lizards (*Uta stansburiana*) at three urban and three rural sites in southwestern Utah, by year

**Figure 6:**
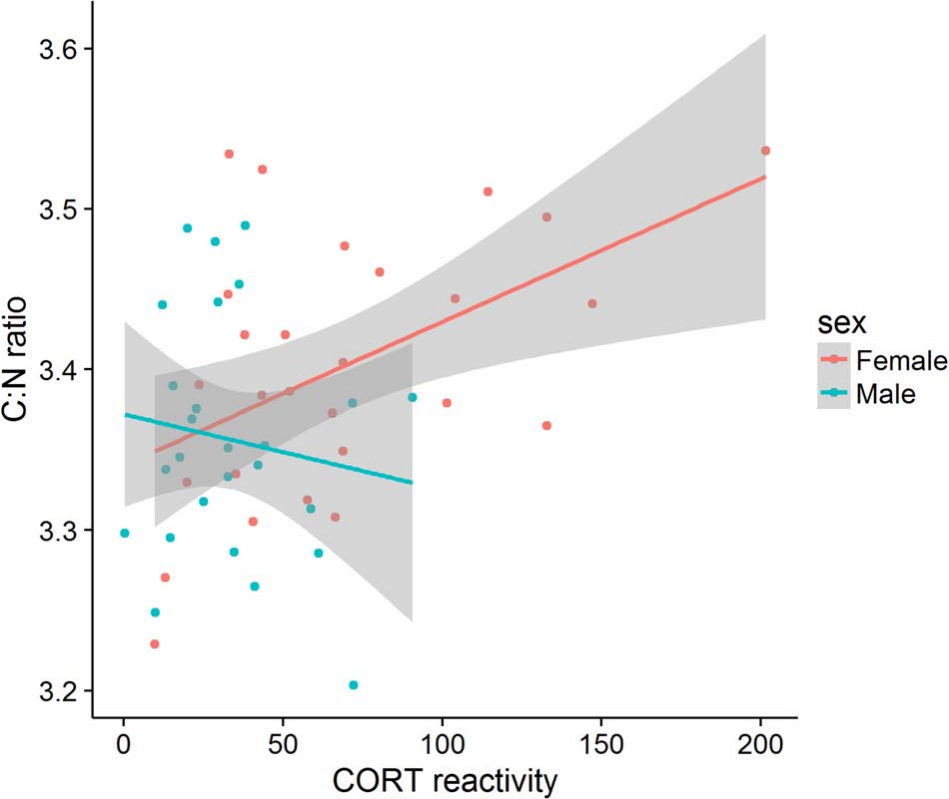
Higher increase in plasma corticosterone after handling in female side-blotched lizards (*Uta stansburiana*) with higher toe C:N ratio in 2013.

In 2013 and 2014, but not in 2015–2017, δ^13^C was related to the total clutch mass of females at urban sites (interaction *F*_7,269_ = 50.8, *P* = 0.0004; *R*^2^ = 0.56; Fig. 5). There was no relationship between δ^15^N or C:N ratio and body condition. In 2013, C:N ratio was positively related to CORT reactivity of female lizards, but not males, at both urban and rural sites (*F*_1,16_ = 12.51, *P* = 0.002; Fig. 6). We did not find significant relationships between isotopic or stoichiometric ratios and other physiological parameters, or significant differences between lizards that were later recaptured and those that were not. We did not find evidence for isotopic differences between sexes or among colour morphs in our populations of *U. stansburiana*.

Lizards at urban and rural sites did not differ significantly in baseline CORT, BKA, OXY, SVL, mass or body condition. Urban animals of both sexes had higher dROMs (*χ*2_1_ = 9.78, *P* < 0.01) (Smith, 2017). There was a strong interaction between urbanization and precipitation, with rural animals increasing their clutch size in wet years and urban animals decreasing (*χ*2_1_ = 16.93, *P* < 0.001). Finally, urban lizards had lower survival than rural ones, (*χ*2_1_ = 8.67, *P* < 0.01), and survival decreased during wet years (*χ*2_1_ = 47.09, *P* < 0.001), with survival of rural animals decreasing more in response to wet years (*χ*2_1_ = 13.44, *P* < 0.01), although overall urban animals were almost six times more likely to die than rural lizards, regardless of precipitation (Smith, 2017).

## Discussion

Across sites, we documented substantial variation in plant, ant and lizard stable isotope signatures. As predicted, we found that there were significant differences in these signatures between rural and urban sites (see map in Fig. S1). However, the relative values of stable isotope ratios were more similar among rural sites than among urban sites and varied over time. We found that stoichiometric and isotopic relationships were related to some physiological parameters (i.e. clutch size in urban animals, stress reactivity), but not to others and these varied over time. Although isotopic variation among colour morphs has been documented in male *Urosaurus ornatus* (Lattanzio and Miles, 2016b), we did not find evidence for such differences in our populations of *U. stansburiana*, which do not exhibit stable colour morphs in southern Utah as they do in other parts of their range (Sinervo and Lively, 1996).

Although populations of *U. stansburiana* appear to be perfectly capable of persisting in even highly altered urban habitats, the life history of *U. stansburiana* (Lucas and French, 2012, Smith *et al.*, 2013) and the occupancy (Smart *et al.*, 2005, Ackley *et al.*, 2015) and physiology (French *et al.*, 2008, French *et al.*, 2010, Knapp *et al.*, 2013) of other lizard species differs between urbanized and rural areas across the globe, a conservation concern that may be partly underpinned by both direct and indirect bottom-up effects of changes to resource quality and availability (Suarez and Case, 2002, Barrett *et al.*, 2005, DeVore and Maerz, 2014, Knapp *et al.*, 2013). We know that habitat deterioration affects body condition, anti-predatory behaviour and parasite loads of lizards (Amo *et al.*, 2007a, Amo *et al.*, 2007b). The use of stable isotope and stoichiometric ratios to assess animal health is a technique that is becoming more widespread (Hatch, 2012). It is a rapid, non-invasive measurement that integrates information over a relatively long time, and the cost is decreasing. However, our test of the generality of relationships between isotopic and stoichiometric ratios and other measures of nutrition and health in a wild reptile revealed that ‘rules’ about the meaning of isotopic and stoichiometric ratios do not apply in all situations (Pilgrim, 2005), and caution should be used when interpreting these data.

### Differences among sites

Sites differed from one another in both δ^13^C and δ^15^N (Fig. 1B); however, it is difficult to ascribe mechanisms to the variation in isotopic signatures across sites. The isotopic structure of food webs is highly variable in space and time (Gannes *et al.*, 1997, Gannes *et al.*, 1998, Pilgrim, 2005), especially in desert ecosystems where patterns of precipitation may be very strong drivers of the isotopic structure of food webs over time via the differential responses of isotopically distinct plant functional groups (Pate and Anson, 2008, Warne *et al.*, 2010). In addition to urbanization, there are pre-existing differences between our urban and rural sites because of chosen human settlement areas. For instance, the percentage of watercourses that are intermittent or ephemeral is higher within 2 km^2^ of our rural sites (58–99%) than at our urban sites (43–49%), largely due to the closer proximity of St. George to the main stem of the perennial Virgin River. The average elevation of rural sites (760–790 m a.s.l.) is also higher than that of urban sites (1180–1240 m a.s.l.), and the historical dominant vegetation differed (Table 2).

The absolute differences we observed among lizards at different sites are driven largely by geographic variation in climate, soil, plants or invertebrates (Smiley *et al.*, 2016; Figs. 1B and 3). The discordance between ant and *U. stansburiana* isotope signatures at urban sites reinforces that *U. stansburiana* feed on many different arthropod species, not only ants, as known from stomach content studies (Knowlton and Nye, 1946, Tinkle, 1967). Furthermore, some authors have suggested inter-individual isotopic variation increases as preferred resources become scarce (Reddin *et al.*, 2016). We found that variation in δ^13^C at urban sites exceeded that at rural sites, which is consistent with the idea that resources at disturbed urban sites may be less optimal than those in relatively undisturbed rural areas. In contrast, other studies have suggested that there may be costs to specialization on high-quality forage (Darimont *et al.*, 2007), such that individuals occupying more peripheral niches have higher fitness. From the perspective of a consumer, the potential differences in nutritional quality and digestibility of C_3_ vs C_4_/CAM plants is partially related to underlying differences in their photosynthetic structures and the resultant difference in C:N ratios, which is one reason why consumers may ‘prefer’ to feed on C_3_ plants (or, in this case, insects that have fed on them) when available (Nagy *et al.*, 1998, Barbehenn *et al.*, 2004, Murray and Wolf, 2013).

Annual variation was present but lesser in magnitude than spatial variation and relatively consistent across sites (Fig. 2). The most divergent year was 2017, in which lizards at all sites were depleted an average of −1.1‰ in δ^13^C compared to other years. This could have been driven by moisture, which was higher in the period leading up to our 2017 sampling than prior to any other year (Table 3). In particular, greater snowpack, the dominant source of most warm-season stream flow in much of Utah (Holmes *et al.*, 1997), ensured stream flow well into May, which we did not observe during any other year. Plants using C_3_ photosynthesis are more ^13^C-depleted than those using C_4_ or CAM (Finlay and Kendall, 2007, Marshall *et al.*, 2007), and these plants may do best in wet years in desert ecosystems, sending a depleted ^13^C signature up the food web to insects (Smith *et al.*, 2002, Spence and Rosenheim, 2005) and their reptilian predators. Even desiccation-resistant reptiles are limited by water in desert ecosystems, altering their activity patterns (with implications for foraging) as well as their geographic distribution (Kearney *et al.*, 2018).

Lattanzio and Miles (2016a) estimated isotopic discrimination of *U. ornatus* claw tissue in the laboratory as δ^13^C = 1.2 ± 0.1 ‰ and δ^15^N = 0.7 ± 0.1 ‰. Differences between ants and *U. stansburiana* whole toes in our wild populations were larger for δ^13^C but similar for δ^15^N at rural sites. Although the toes that we used included claw tissue, the whole toe would essentially be analogous to whole body tissue in that it is integrating isotopic ratios over the life of the animal. This is an important difference; however, no more appropriate isotopic discrimination factors have been developed. Additionally, because each lizard had a unique toe clip code, a different combination of one to four toes was collected from every individual, but we expect this variation to be random with respect to isotopic composition. Assuming that these discrimination values are approximately correct, our data suggest that ants may contribute to the diet of wild *U. stansburiana* in rural areas, which is consistent with results from more directed studies of diet conducted in rural ecosystems (Tinkle, 1967, Best and Gennaro, 1984), although *U. stansburiana* are not ant specialists (Knowlton and Nye, 1946). However, estimating absolute or relative dietary contributions of various food sources relies on the assumption that all of the potential food sources are sampled and analyzed, which is not the case nor the intent here.

The diet of *U. stansburiana* in urban areas has not been studied, but our data suggest that it may vary substantially from that in rural areas. In particular, we suggest that urban *U. stansburiana* diet is more varied, because the stable isotopes signatures of *U. stansburiana* toes at urban sites exhibited high inter-individual variation than those at rural sites. Alternatively, *U. stansburiana* at urban and rural sites may be feeding on similar invertebrates that draw their nutrition from a wider variety of plant resources. Because *Pogonomyrmex* ants at urban sites did not have stable isotope signatures that were depleted relative to *U. stansburiana* toes, we suggest that *U. stansburiana* are probably not feeding on these ants at urban sites. Aegean wall lizards (*Podarcis erhardii*) use different foraging modes and thus differ in diet between urban and rural environments (Donihue, 2016), and even ant-specialist lizards such as *Phrynosoma* shift their diets and select the most profitable available prey in urban environments that lack preferred food sources (Ramakrishnan *et al.*, 2018).

At urban sites, *U. stansburiana* had carbon signatures that were more similar to those of C_4_ rather than C_3_ plants. This was surprising because the dominant vegetation surrounding two of our rural sites was 18–45% made up of C_4_*Atriplex confertifolia* (Table 2; Utah Automated Geographic Reference Center, 2019). However, a substantial proportion of primary producer biomass in these systems is herbaceous vegetation that is not accounted for in dominant vegetation classifications. Although our urban sites included a mixture of C_3_ and C_4_ plants, three elements associated with urbanization could lead to increased C_4_ food web contributions in urban areas: (i) non-native C_4_ plant invasion (Ehrenfeld, 2003, Bradford *et al.*, 2010), (ii) agriculture of C_4_ plants such as corn (Finucane *et al.*, 2006) and (iii) human detritus, such as food waste (Schoeller *et al.*, 1986, Jahren *et al.*, 2014), fed on by invertebrates. All of our sites are relatively distant from agriculture (and, the most commonly grown crop in Washington County is alfalfa, a C_3_ legume). The most common non-native C_4_ plant is *Kochia*. Smith *et al.* (2002) suggested that resource selection by arthropods is tied to the production of C_3_ plants and that lags of 1–2 years in the passage of carbon up the food chain may exist in desert ecosystems. Although individual *Pogonomyrmex* worker ants only live about 30 days as larvae (Willard and Crowell, 1965) and 15–30 days as adults (Gordon and Hölldobler, 1987), colonies may persist for 20–40 years (Keeler, 1982), and seeds are stored for later consumption (MacKay, 1985, Smith, 2007). Testing these hypotheses requires more consistent sampling that would allow the creation of more complete food webs.

Our sample sizes for prey are relatively small and do not completely cover the temporal span of lizard tissue samples, which means it may be difficult to directly compare them. However (with the exception of site U2, which was developed in late 2015), we did not observe evidence of major habitat alteration or plant community change throughout the study period either during field work or from Google Earth imagery.

### Co-variation with physiology

We present the first evidence that reproductive physiological demands (clutch size) and proximate physiological mechanisms underlying energy allocation (CORT reactivity) can co-vary with stoichiometric or stable isotope ratios, possibly as a result of variation in nutritional stress. In cases of resource limitation, trade-offs among competing demands often emerge.

We found that higher CORT reactivity to a controlled stressor was associated with higher C:N ratios in some cases, suggesting that animals in superior body condition that likely had access to ample lipid reserves may also be more capable of mobilizing stored energy (French *et al.*, 2007, Price, 2017). This co-variation is likely underlain by variation in nutritional status, although we did not detect any relationship between C:N or δ^15^N and measures of nutritional status such as body condition. Immune state is associated with natural dietary variation in wild mice (Taylor *et al.*, 2019), but we did not find any evidence that innate immunity of lizards at our sites co-varied with C:N, δ^13^C, or δ^15^N.

Although the primary sources of mortality in our system are still unknown, nutritional constraints likely influence several sources of mortality. Although Wilson and Cooke (2004) found no relationship between overwinter survival and body condition or body mass of *U. stansburiana*, liver glycogen, not lipid, limitation, was implicated as the primary cause of mortality in overwintering *U. stansburiana* in the lab (Zani *et al.*, 2012) and lipid metabolism at low temperatures may be arrested. Animals with insufficient dietary energetic resources first metabolize lipids and only as a last resort catabolize protein for energy (McCue *et al.*, 2013, McCue *et al.*, 2015a, McCue *et al.*, 2015b). Lipid catabolism without replenishment of lipid stores may cause changes in the δ^13^C of animal tissues, whereas protein catabolism may cause changes in the δ^15^N signature of animal tissues; both may induce shifts in body stoichiometry (C:N ratio; via increases in C-rich carbohydrates and reductions in N-rich proteins; Zhang *et al.*, 2016). Lab studies suggest that these two phases of nutritional stress rarely overlap; that is, starving animals spare protein until lipid reserves are exhausted (McCue, 2010). Thus, future studies should examine the predictive value of isotopic and stoichiometric ratios on survival probability and attempt to unravel the mechanisms by which nutritional status impacts survival.

Urban lizards had smaller clutches in wet years (Smith, 2017), which could be due to influences of urbanization, elevation or both on thermoregulation, nutrition or other aspects of *U. stansburiana* ecology. Although mechanisms in this system remain unclear, rainfall (used as a surrogate for ecosystem productivity), together with latitude, is an important determinant of population density and home-range size of *U. stansburiana* at a continental scale (Scoular *et al.*, 2011). Female lizards with larger clutches at urban sites probably increased in δ^13^C as a result of the larger nutritional (lipid) cost of vitellogenesis (Fuller *et al.*, 2004, Hatch, 2012). We observed this relationship only at urban sites. It would be worthwhile to examine differences in lipid storage capacity of female *U. stansburiana* in rural and urbanized habitats. Because overwinter mortality is significant in this species (Tinkle, 1967, Zani, 2005), there could be strong fitness effects resulting from allocation of lipids to vitellogenesis (Price, 2017). Furthermore, this effect was strongest in the two driest years (Table 3), so it may manifest only below certain thresholds of nutrient limitation.

### Reptiles in ecosystems

Within ecosystems, reptiles can reach high densities (Rodda *et al.*, 2001, Novosolov *et al.*, 2018) and may represent large standing stocks of nitrogen and other limiting nutrients (Milanovich *et al.*, 2015). Studies of their stoichiometry are in their infancy (Sterrett *et al.*, 2015). In particular, the excreta of many tetrapods represent a contribution of organic forms of nitrogen (urea or uric acid) rather than inorganic ammonia (excreted by fishes), thereby providing both carbon and nitrogen to microbes and potentially representing an important and overlooked mechanism of nutrient recycling of limiting nutrients in many ecosystems (Milanovich *et al.*, 2015, Milanovich and Hopton, 2016, Milanovich and Peterman, 2016).

Interest in the beneficial roles of reptiles in ecosystems is old (Knowlton, 1934), but few studies have directly addressed this topic. Reptiles are becoming increasingly better-understood models for ecological, behavioural and physiological research (Shine and Bonnet, 2000, Blackburn, 2006). As urbanization continues to impact wildlife populations, we stand to lose both biodiversity and probably unknown and overlooked ecosystem functions (Gibbons *et al.*, 2000, Willson and Winne, 2016). Stoichiometric and stable isotope ratios can provide unique insights into the mechanisms and linkages underlying the profound effects that urbanization can have on wild animal ecology and physiology.

## Conclusions

We showed that annual and spatial co-variation in stable isotope signatures of plants, ants and lizards exists in an arid Mojave Desert ecosystem. Some annual variation is likely driven by precipitation, whereas spatial variation is extensive, though influenced by mechanisms that are not yet clear.

## Supplementary Material

wildUtapaper_ConsPhys_r2_coaa001

## References

[ref1] Ackley JW, Wu J, Angilletta MJ Jr, Myint SW, Sullivan B (2015) Rich lizards: how affluence and land cover influence the diversity and abundance of desert reptiles persisting in an urban landscape. Biol Conserv 182: 87–92.

[ref2] Amo L, López P, Martı J (2006) Nature-based tourism as a form of predation risk affects body condition and health state of *Podarcis muralis* lizards. Biol Conserv 131: 402–409.

[ref3] Amo L, Lopez P, Martin J (2007a) Habitat deterioration affects antipredatory behavior, body condition, and parasite load of female *Psammodromus algirus* lizards. Can J Zool 85: 743–751.

[ref4] Amo L, Lopez P, Martín J (2007b) Habitat deterioration affects body condition of lizards: a behavioral approach with *Iberolacerta cyreni* lizards inhabiting ski resorts. Biol Conserv 135: 77–85.

[ref5] Audsley BW, Bock CE, Jones ZF, Bock JH, Smith HM (2006) Lizard abundance in an exurban southwestern savanna, and the possible importance of roadrunner predation. Am Midl Nat 155: 395–402.

[ref6] Barbehenn RV, Karowe DN, Chen Z (2004) Performance of a generalist grasshopper on a C_3_ and a C_4_ grass: compensation for the effects of elevated CO_2_ on plant nutritional quality. Oecologia 140: 96–103. 15069636 10.1007/s00442-004-1555-x

[ref7] Barrett K, Anderson WB, Wait DA, Grismer LL (2005) Marine subsidies alter the diet and abundance of insular and coastal lizard populations. Oikos 109: 145–153.

[ref8] Best TL, Gennaro A (1984) Feeding ecology of the lizard, *Uta stansburiana*, in southeastern New Mexico. J Herpetol 18: 291–301.

[ref9] Blackburn DG (2006) Squamate reptiles as model organisms for the evolution of viviparity. Herpetol Monog 20: 131–146.

[ref10] Bradford MA, DeVore JL, Maerz JC, McHugh JV, Smith CL, Strickland MS (2010) Native, insect herbivore communities derive a significant proportion of their carbon from a widespread invader of forest understories. Biol Invasions 12: 721–724.

[ref11] Bradley CA, Altizer S (2007) Urbanization and the ecology of wildlife diseases. Trends Ecol Evol 22: 95–102. 17113678 10.1016/j.tree.2006.11.001 PMC7114918

[ref12] Comendant T, Sinervo B, Svensson E, Wingfield J (2003) Social competition, corticosterone and survival in female lizard morphs. J Evol Biol 16: 948–955. 14635910 10.1046/j.1420-9101.2003.00598.x

[ref13] Darimont C, Paquet P, Reimchen T (2007) Stable isotopic niche predicts fitness of prey in a wolf–deer system. Biol J Linn Soc 90: 125–137.

[ref14] DeVore JL, Maerz JC (2014) Grass invasion increases top-down pressure on an amphibian via structurally mediated effects on an intraguild predator. Ecology 95: 1724–1730. 25163106 10.1890/13-1715.1

[ref15] Dickens MJ, Romero LM (2013) A consensus endocrine profile for chronically stressed wild animals does not exist. Gen Comp Endocr 191: 177–189. 23816765 10.1016/j.ygcen.2013.06.014

[ref16] Ditchkoff SS, Saalfeld ST, Gibson CJ (2006) Animal behavior in urban ecosystems: modifications due to human-induced stress. Urban Ecosyst 9: 5–12.

[ref17] Donihue CM (2016) Aegean wall lizards switch foraging modes, diet, and morphology in a human-built environment. Ecol Evol 6: 7433–7442. 28725410 10.1002/ece3.2501 PMC5513264

[ref18] Durso AM, Seigel RA (2015) A snake in the hand is worth 10,000 in the bush. J Herpetol 49: 503–506.

[ref19] Ehrenfeld JG (2003) Effects of exotic plant invasions on soil nutrient cycling processes. Ecosystems 6: 503–523.

[ref20] Finlay JC, Kendall C (2007) Stable isotope tracing of temporal and spatial variability in organic matter sources to freshwater ecosystems In Michener R, Lajtha K, eds, Stable Isotopes in Ecology and Environmental Science, Ed 2nd. Blackwell Publishing, Singapore, pp. 283–333.

[ref21] Finucane B, Agurto PM, Isbell WH (2006) Human and animal diet at Conchopata, Peru: stable isotope evidence for maize agriculture and animal management practices during the Middle Horizon. J Archaeol Sci 33: 1766–1776.

[ref22] Fox J, Weisberg S (2011) An {R} Companion to Applied Regression, EdEd 2 Sage, Thousand Oaks.

[ref23] French SS, DeNardo DF, Greives TJ, Strand CR, Demas GE (2010) Human disturbance alters endocrine and immune responses in the Galapagos marine iguana (*Amblyrhynchus cristatus*). Horm Behav 58: 792–799. 20708010 10.1016/j.yhbeh.2010.08.001 PMC2982938

[ref24] French SS, DeNardo DF, Moore MC (2007) Trade-offs between the reproductive and immune systems: facultative responses to resources or obligate responses to reproduction? Am Nat 170: 79–89. 17853993 10.1086/518569

[ref25] French SS, Fokidis HB, Moore MC (2008) Variation in stress and innate immunity in the tree lizard *(Urosaurus ornatus*) across an urban–rural gradient. J Comp Physiol B 178: 997–1005. 18594834 10.1007/s00360-008-0290-8 PMC2774757

[ref26] French SS, Neuman-Lee LA (2012) Improved *ex vivo* method for microbiocidal activity across vertebrate species. Biol Open 1: 482–487. 23213440 10.1242/bio.2012919 PMC3507210

[ref27] French SS, Webb AC, Hudson SB, Virgin EE (2018) Town and country reptiles: a review of reptilian responses to urbanization. Integr Comp Biol 58: 948–966. 29873730 10.1093/icb/icy052

[ref28] Fuller BT, Fuller JL, Sage NE, Harris DA, O'Connell TC, Hedges RE (2004) Nitrogen balance and δ^15^N: why you’re not what you eat during pregnancy. Rapid Commun Mass Sp 18: 2889–2896. 10.1002/rcm.1708 15517531

[ref29] Gannes LZ, Martinez del Rio C, Koch P (1998) Natural abundance variations in stable isotopes and their potential uses in animal physiological ecology. Comp Biochem Phys A 119: 725–737. 10.1016/s1095-6433(98)01016-2 9683412

[ref30] Gannes LZ, O'Brien DM, Martinez del Rio C (1997) Stable isotopes in animal ecology: assumptions, caveats, and a call for more laboratory experiments. Ecology 78: 1271–1276.

[ref31] Gibbons JW, Scott DE, Ryan TJ, Buhlmann KA, Tuberville TD, Metts BS, Greene JL, Mills T, Leiden Y, Poppy S (2000) The global decline of reptiles, déjà vu amphibians. Bioscience 50: 653–666.

[ref32] Glanville E, Seebacher F (2006) Compensation for environmental change by complementary shifts of thermal sensitivity and thermoregulatory behaviour in an ectotherm. J Exp Biol 209: 4869–4877. 17142675 10.1242/jeb.02585

[ref33] Gordon DM, Hölldobler B (1987) Worker longevity in harvester ants (*Pogonomyrmex*). Psyche J Entomol 94: 341–346.

[ref34] Graves GR, Newsome SD, Willard DE, Grosshuesch DA, Wurzel WW, Fogel ML (2012) Nutritional stress and body condition in the Great Gray Owl (*Strix nebulosa*) during winter irruptive migrations. Can J Zool 90: 787–797.

[ref35] Hartman G (2011) Are elevated delta ^15^N values in herbivores in hot and arid environments caused by diet or animal physiology? Funct Ecol 25: 122–131.

[ref36] Hatch KA (2012) The use and application of stable isotope analysis to the study of starvation, fasting, and nutritional stress in animals In McCue MD, ed, Comparative Physiology of Fasting, Starvation, and Food Limitation. Springer-Verlag, Berlin, pp. 337–364.

[ref37] Hayashi I (1983) Seasonal changes in condition factors and in the C:N ratio of the foot of the ormer, *Haliotis tuberculata*. J Mar Biol Assoc U K 63: 85–95.

[ref38] Holmes WF, Pyper GE, Gates JS, Schaefer DH, Waddell KM (1997) Hydrology and water quality of the Beaver Dam Wash area, Washington County, Utah, Lincoln County, Nevada, and Mohave County, Arizona In US Geological Survey Water Resources Investigations Report. US Geological Survey.

[ref39] Hopkins WA (2007) Amphibians as models for studying environmental change. ILAR J 48: 270–277. 17592188 10.1093/ilar.48.3.270

[ref40] Jahren AH, Bostic JN, Davy BM (2014) The potential for a carbon stable isotope biomarker of dietary sugar intake. J Anal Atom Spectrom 29: 795–816.

[ref41] Kattge J et al. (2011) TRY – a global database of plant traits. Glob Change Biol 17: 2905–2935.

[ref42] Kearney MR, Munns SL, Moore D, Malishev M, Bull CM (2018) Field tests of a general ectotherm niche model show how water can limit lizard activity and distribution. Ecol Monogr 88: 672–693.

[ref43] Keeler KH (1982) Preliminary report of colony survivorship in the Western Harvester Ant (*Pogonomyrmex occidentalis*) in Western Nebraska. Southwest Nat 27: 245–246.

[ref44] Knapp CR, Hines KN, Zachariah TT, Perez-Heydrich C, Iverson JB, Buckner SD, Halach SC, Lattin CR, Romero LM (2013) Physiological effects of tourism and associated food provisioning in an endangered iguana. Conserv Physiol 1: 1–12. 10.1093/conphys/cot032 PMC4806617 27293616

[ref45] Knowlton GF (1934) Lizards as a factor in the control of range insects. J Econ Entomol 27: 998–1004.

[ref46] Knowlton GF, Nye WP (1946) Lizards feeding on ants in Utah. J Econ Entomol 39: 546.21000985

[ref47] Krebs J, Lofroth E, Copeland J, Banci V, Cooley D, Golden H, Magoun A, Mulders R, Shults B (2004) Synthesis of survival rates and causes of mortality in North American wolverines. J Wildl Manag 68: 493–502.

[ref48] Lattanzio M, Miles D (2016a) Stable carbon and nitrogen isotope discrimination and turnover in a small-bodied insectivorous lizard. Isot Environ Health S 52: 673–681. 10.1080/10256016.2016.1154854 26999652

[ref49] Lattanzio MS, Miles DB (2016b) Trophic niche divergence among colour morphs that exhibit alternative mating tactics. Roy Soc Open Sci 3: 150531. 27152203 10.1098/rsos.150531 PMC4852626

[ref50] Lucas L, French SS (2012) Stress-induced tradeoffs in a free-living lizard across a variable landscape: consequences for individuals and populations. PLoS ONE 7: e49895. 23185478 10.1371/journal.pone.0049895 PMC3502225

[ref51] MacKay WP (1985) A comparison of the energy budgets of three species of *Pogonomyrmex* harvester ants (Hymenoptera: Formicidae). Oecologia 66: 484–494. 28310787 10.1007/BF00379338

[ref52] Marshall JD, Brooks JR, Lajtha K (2007) Sources of variation in the stable isotopic composition of plants In Michener R, Lajtha K, eds, Stable Isotopes in Ecology and Environmental Science, Ed 2 Blackwell Publishing, Singapore, pp 22–60.

[ref53] Martinez del Rio C, Wolf N, Carleton SA, Gannes LZ (2009) Isotopic ecology ten years after a call for more laboratory experiments. Biol Rev 84: 91–111. 19046398 10.1111/j.1469-185X.2008.00064.x

[ref54] McCue MD (2010) Starvation physiology: reviewing the different strategies animals use to survive a common challenge. Comp Biochem Physiology A Mol Integr Physiol 156: 1–18. 10.1016/j.cbpa.2010.01.002 20060056

[ref55] McCue MD, Amaya JA, Yang AS, Erhardt EB, Wolf BO, Hanson DT (2013) Targeted ^13^C enrichment of lipid and protein pools in the body reveals circadian changes in oxidative fuel mixture during prolonged fasting: a case study using Japanese quail. Comp Biochem Physiol A Mol Integr Physiol 166: 546–554. 23988480 10.1016/j.cbpa.2013.08.009

[ref56] McCue MD, Guzman RM, Passement CA (2015a) Digesting pythons quickly oxidize the proteins in their meals and save the lipids for later. J Exp Biol 215: 2089–2096. 10.1242/jeb.118349 25987734

[ref57] McCue MD, Guzman RM, Passement CA, Davidowitz G (2015b) How and when do insects rely on endogenous protein and lipid resources during lethal bouts of starvation? A new application for ^13^C-breath testing. PLoS ONE 10: e0140053. 26465334 10.1371/journal.pone.0140053 PMC4605643

[ref58] McCue MD, Pollock ED (2008) Stable isotopes may provide evidence for starvation in reptiles. Rapid Commun Mass Spectrom 22: 2307–2314. 18613003 10.1002/rcm.3615

[ref59] McKinney ML (2008) Effects of urbanization on species richness: a review of plants and animals. Urban Ecosyst 11: 161–176.

[ref60] McNamara JM, Houston AI (1990) The value of fat reserves and the tradeoff between starvation and predation. Acta Biotheor 38: 37–61. 2109917 10.1007/BF00047272

[ref61] Mekota AM, Grupe G, Ufer S, Cuntz U (2006) Serial analysis of stable nitrogen and carbon isotopes in hair: monitoring starvation and recovery phases of patients suffering from anorexia nervosa. Rapid Commun Mass Spectrom 20: 1604–1610. 16628564 10.1002/rcm.2477

[ref62] Milanovich JR, Hopton ME (2016) Stoichiometry of excreta and excretion rates of a stream-dwelling plethodontid salamander. Copeia 104: 26–34.

[ref63] Milanovich JR, Maerz JC, Rosemond AD (2015) Stoichiometry and estimates of nutrient standing stocks of larval salamanders in Appalachian headwater streams. Freshw Biol 60: 1340–1353.

[ref64] Milanovich JR, Peterman WE (2016) Revisiting Burton and Likens (1975): nutrient standing stock and biomass of a terrestrial salamander in the Midwestern United States. Copeia 104: 165–171.

[ref65] Mills SC, Hazard L, Lancaster L, Mappes T, Miles D, Oksanen TA, Sinervo B (2008) Gonadotropin hormone modulation of testosterone, immune function, performance, and behavioral trade-offs among male morphs of the lizard *Uta stansburiana*. Am Nat 171: 339–357. 18201140 10.1086/527520

[ref66] Mitchell JC, Jung RE, Bartholomew B (2008) Urban Herpetology. Society for the Study of Amphibians and Reptiles, Salt Lake City.

[ref67] Monteith KL, Bleich VC, Stephenson TR, Pierce BM, Conner MM, Kie JG, Bowyer RT (2014) Life-history characteristics of mule deer: effects of nutrition in a variable environment. Wildl Monog 186: 1–62.

[ref68] Moore MC, Thompson CW, Marler CA (1991) Reciprocal changes in corticosterone and testosterone levels following acute and chronic handling stress in the tree lizard, *Urosaurus ornatus*. Gen Comp Endocrinol 81: 217–226. 2019396 10.1016/0016-6480(91)90006-r

[ref69] Murray IW, Wolf BO (2013) Desert tortoise (*Gopherus agassizii*) dietary specialization decreases across a precipitation gradient. PLoS ONE 8: e66505. 23840495 10.1371/journal.pone.0066505 PMC3696026

[ref70] Murray M, Cembrowski A, Latham A, Lukasik V, Pruss S, St Clair C (2015) Greater consumption of protein-poor anthropogenic food by urban relative to rural coyotes increases diet breadth and potential for human–wildlife conflict. Ecography 38: 1235–1242.

[ref71] Nagy KA, Henen BT, Vyas DB (1998) Nutritional quality of native and introduced food plants of wild desert tortoises. J Herpetol 32: 260–267.

[ref72] National Oceanic and Atmospheric Administration (2018). Global Historical Climatology Network Daily.

[ref73] Naug D (2009) Nutritional stress due to habitat loss may explain recent honeybee colony collapses. Biol Conserv 142: 2369–2372.

[ref74] Neuman-Lee LA, Stokes AN, Greenfield S, Hopkins GR, Brodie ED, French SS (2015) The role of corticosterone and toxicity in the antipredator behavior of the Rough-skinned Newt (*Taricha granulosa*). Gen Comp Endocrinol 213: 59–64. 25556312 10.1016/j.ygcen.2014.12.006

[ref75] Novosolov M, Rodda GH, Gainsbury AM, Meiri S (2018) Dietary niche variation and its relationship to lizard population density. J Anim Ecol 87: 285–292. 28944457 10.1111/1365-2656.12762

[ref76] Okumura T, Nagasawa T, Hayashi I, Sato Y (2002) Effects of starvation on RNA: DNA ratio, glycogen content, and C:N ratio in columellar muscle of the Japanese turban shell Turbo (*Batillus*) cornutus (Gastropoda). Fish Sci 68: 306–312.

[ref77] Parnell A, Jackson A (2011). SIAR: stable isotope analysis in R. R package version 4.2.

[ref78] Pate FD, Anson TJ (2008) Stable nitrogen isotope values in arid-land kangaroos correlated with mean annual rainfall: potential as a palaeoclimatic indicator. Int J Osteoarchaeol 18: 317–326.

[ref79] Pilgrim M (2005) Linking microgeographic variation in pigmy rattlesnake (Sistrurus miliarius) life history and demography with diet composition: a stable isotope approach In Ph. D. Ph. D. Dissertation. University of Arkansas, Fayetteville.

[ref80] Pinheiro J, Bates D, DebRoy S, Sarkar D, R Core Team (2016). Nlme: linear and nonlinear mixed effects models, Ed R package version 3.1-128.

[ref81] Post DM (2002) Using stable isotopes to estimate trophic position: models, methods, and assumptions. Ecology 83: 703–718.

[ref82] Post DM, Layman CA, Arrington DA, Takimoto G, Quattrochi J, Montana CG (2007) Getting to the fat of the matter: models, methods and assumptions for dealing with lipids in stable isotope analyses. Oecologia 152: 179–189. 17225157 10.1007/s00442-006-0630-x

[ref83] Price ER (2017) The physiology of lipid storage and use in reptiles. Biol Rev 92: 1406–1426. 27348513 10.1111/brv.12288

[ref84] Core Team R (2019) R: A Language and Environment for Statistical Computing. R Foundation for Statistical Computing, Vienna, Austria.

[ref85] Ramakrishnan S, Wolf AJ, Hellgren EC, Moody RW, Bogosian V (2018) Diet selection by a lizard ant-specialist in an urban system bereft of preferred prey. J Herpetol 52: 79–85.

[ref86] Reddin CJ, O’Connor NE, Harrod C (2016) Living to the range limit: consumer isotopic variation increases with environmental stress. Peer J 4: e2034. 27280067 10.7717/peerj.2034 PMC4893340

[ref87] Refsnider JM, Janzen FJ (2012) Behavioural plasticity may compensate for climate change in a long-lived reptile with temperature-dependent sex determination. Biol Conserv 152: 90–95.

[ref88] Rodda GH, Dean-Bradley K, Campbell EW, Fritts TH, Lardner B, Yackel Adams AA, Reed RN (2015) Stability of detectability over 17 years at a single site and other lizard detection comparisons from Guam. J Herpetol 49: 513–521.

[ref89] Rodda GH, Perry GAD, Rondeau RJ, Lazell J (2001) The densest terrestrial vertebrate. J Trop Ecol 17: 331–338.

[ref90] Roeder KA, Kaspari M (2017) From cryptic herbivore to predator: stable isotopes reveal consistent variability in trophic levels in an ant population. Ecology 98: 297–303. 28052342 10.1002/ecy.1641

[ref91] Romero LM, Reed JM (2005) Collecting baseline corticosterone samples in the field: is under 3 min good enough? Comp Biochem Phys A Mol Integr Phys 140: 73–79. 10.1016/j.cbpb.2004.11.004 15664315

[ref92] Romero LM, Reed JM (2008) Repeatability of baseline corticosterone concentrations. Gen Comp Endocrinol 156: 27–33. 18036526 10.1016/j.ygcen.2007.10.001

[ref93] Romero LM, Wikelski M (2001) Corticosterone levels predict survival probabilities of Galapagos marine iguanas during El Niño events. Proc Natl Acad Sci 98: 7366–7370. 11416210 10.1073/pnas.131091498 PMC34674

[ref94] Romero LM, Wikelski M (2010) Stress physiology as a predictor of survival in Galapagos marine iguanas. P Royal Soc London B Biol Sci 277: 3157–3162. 10.1098/rspb.2010.0678 PMC2982063 20504812

[ref95] Sarre S, Dearn JM, Georges A (1994) The application of fluctuating asymmetry in the monitoring of animal populations. Pac Conserv Biol 1: 118–122.

[ref96] Schoeller DA, Minagawa M, Slater R, Kaplan I (1986) Stable isotopes of carbon, nitrogen and hydrogen in the contemporary North American human food web. Ecology of Food and Nutrition 18: 159–170.

[ref97] Scoular KM, Caffry WC, Tillman JL, Finan ES, Schwartz SK, Sinervo B, Zani PA (2011) Multiyear home-range ecology of common side-blotched lizards in eastern Oregon with additional analysis of geographic variation in home-range size. Herpetological Monographs 25: 52–75.

[ref98] Shine R, Bonnet X (2000) Snakes: a new 'model organism' in ecological research? Trends in Ecology & Evolution 15: 221–222. 10802545 10.1016/s0169-5347(00)01853-x

[ref99] Sinervo B, Lively CM (1996) The rock-paper-scissors game and the evolution of alternative male strategies. Nature 380: 240–243.

[ref100] Smart R, Whiting MJ, Twine W (2005) Lizards and landscapes: integrating field surveys and interviews to assess the impact of human disturbance on lizard assemblages and selected reptiles in a savanna in South Africa. Biological Conservation 122: 23–31.

[ref101] Smiley TM, Cotton JM, Badgley C, Cerling TE (2016) Small-mammal isotope ecology tracks climate and vegetation gradients across western North America. Oikos 125: 1100–1109.

[ref102] Smith CR (2007) Energy use and allocation in the Florida harvester ant, *Pogonomyrmex badius*: are stored seeds a buffer? Behav Ecol Sociobiol 61: 1479–1487.

[ref103] Smith GD, Lucas LD, French SS (2013) Town and country lizards: physiological ecology of Side-blotched Lizards across a variable landscape In Lutterschmidt WI, ed, Reptiles in Research. Nova Biomedical, New York, pp 51–71.

[ref104] Smith GDS (2017) Natural and Anthropogenic Effects on Life History Characteristics in the Side-blotched Lizard (*Uta stansburiana*). PhD dissertation. Utah State University, Logan.

[ref105] Smith K, Sharp Z, Brown J (2002) Isotopic composition of carbon and oxygen in desert fauna: investigations into the effects of diet, physiology, and seasonality. J Arid Environ 52: 419–430.

[ref106] Speakman JR (2008) Body Composition Analysis of Animals: A Handbook of Non-destructive Methods. Cambridge University Press, Cambridge, p. 260.

[ref107] Spence KO, Rosenheim JA (2005) Isotopic enrichment in herbivorous insects: a comparative field-based study of variation. Oecologia 146: 89–97. 16012818 10.1007/s00442-005-0170-9

[ref108] Sterrett SC, Maerz JC, Katz RA (2015) What can turtles teach us about the theory of ecological stoichiometry? Freshw Biol 60: 443–455.

[ref109] Stout IJ, Cornwell GW (1976) Nonhunting mortality of fledged North American waterfowl. J Wildl Manag 40: 681–693.

[ref110] Suarez AV, Case TJ (2002) Bottom-up effects on persistence of a specialist predator: ant invasions and horned lizards. Ecol Appl 12: 291–298.

[ref111] Suorsa P, Helle H, Koivunen V, Huhta E, Nikula A, Hakkarainen H (2004) Effects of forest patch size on physiological stress and immunocompetence in an area-sensitive passerine, the Eurasian treecreeper (*Certhia familiaris*): an experiment. Proc Roy Soc B Biol Sci 271: 435–440. 10.1098/rspb.2003.2620 PMC1691602 15101703

[ref112] Taylor CH, Young S, Fenn J, Lamb AL, Lowe AE, Poulin B, MacColl ADC, Bradley JE (2019) Immune state is associated with natural dietary variation in wild mice *Mus musculus domesticus*. Funct Ecol 33: 1425–1435. 31588159 10.1111/1365-2435.13354 PMC6767599

[ref113] Tillberg CV, McCarthy DP, Dolezal AG, Suarez AV (2006) Measuring the trophic ecology of ants using stable isotopes. Insectes Sociaux 53: 65–69.

[ref114] Tinkle DW (1967) The life and demography of the side-blotched lizard, *Uta stansburiana*. Miscellaneous Publications of the Museum of Zoology University of Michigan 132: 1–182.

[ref115] U. S. Census Bureau (2010) Census Blocks with Population and Housing Counts. https://www.census.gov/geographies/mapping-files/time-series/geo/tiger-line-file.2010.html.

[ref116] U. S. Census Bureau (2019) Census Bureau Release Number CB19–55. https://www.census.gov/newsroom/press-releases/2019/estimates-county-metro.html.

[ref117] Utah Automated Geographic Reference Center (2019) Utah State Geographic Information Database. gis.utah.gov.

[ref118] Vangestel C, Braeckman BP, Matheve H, Lens L (2010) Constraints on home range behaviour affect nutritional condition in urban house sparrows (*Passer domesticus*). Biol J Linn Soc 101: 41–50.

[ref119] Warne RW, Pershall AD, Wolf BO (2010) Linking precipitation and C_3_–C_4_ plant production to resource dynamics in higher-trophic-level consumers. Ecology 91: 1628–1638. 20583705 10.1890/08-1471.1

[ref120] Wickham H (2009) ggplot2: Elegant Graphics for Data Analysis. Springer-Verlag, New York.

[ref121] Wikelski M, Romero LM (2003) Body size, performance and fitness in Galapagos marine iguanas. Integr Comp Biol 43: 376–386. 21680446 10.1093/icb/43.3.376

[ref122] Willard JR, Crowell HH (1965) Biological activities of the harvester ant, *Pogonomyrmex owyheei*, in Central Oregon. J Econ Entomol 58: 484–489.

[ref123] Willson JD, Winne CT (2016) Evaluating the functional importance of secretive species: a case study of aquatic snake predators in isolated wetlands. J Zool 298: 266–273.

[ref124] Willson JD, Winne CT, Dorcas ME, Gibbons JW (2006) Post-drought responses of semi-aquatic snakes inhabiting an isolated wetland: insights on different strategies for persistence in a dynamic habitat. Wetlands 26: 1071–1078.

[ref125] Willson JD, Winne CT, Pilgrim MA, Romanek CS, Gibbons JW (2010) Seasonal variation in terrestrial resource subsidies influences trophic niche width and overlap in two aquatic snake species: a stable isotope approach. Oikos 119: 1161–1171.

[ref126] Wilson BS, Cooke DE (2004) Latitudinal variation in rates of overwinter mortality in the lizard *Uta stansburiana*. Ecology 85: 3406–3417.

[ref127] Young TP (1994) Natural die-offs of large mammals: implications for conservation. Conserv Biol 8: 410–418.

[ref128] Zani PA (2005) Life-history strategies near the limits of persistence: winter survivorship and spring reproduction in the common side-blotched lizard (*Uta stansburiana*) in eastern Oregon. J Herpetol 39: 166–169.

[ref129] Zani PA, Irwin JT, Rollyson ME, Counihan JL, Healas SD, Lloyd EK, Kojanis LC, Fried B, Sherma J (2012) Glycogen, not dehydration or lipids, limits winter survival of side-blotched lizards (*Uta stansburiana*). J Exp Biol 215: 3126–3134. 22875774 10.1242/jeb.069617

[ref130] Zhang C, Jansen M, De Meester L, Stoks R (2016) Energy storage and fecundity explain deviations from ecological stoichiometry predictions under global warming and size-selective predation. J Anim Ecol 85: 1431–1441. 27080908 10.1111/1365-2656.12531

