# Supplementary material for: Stoichiometric and stable isotope ratios of wild lizards in an urban landscape vary with reproduction, physiology, space and time
Source: Conserv Physiol. 2020 Feb 14;8(1):coaa001. doi: 10.1093/conphys/coaa001 (PMC7019090; doi:10.1093/conphys/coaa001)
Supplement: wildUtapaper_ConsPhys_r2_coaa001 [file wildutapaper_consphys_r2_coaa001.docx]

Figure S1: Map of the six sites, showing human population density, roads, streams, and municipalities. GIS data from Utah Automated Geographic Reference Center (2019) and U. S. Census Bureau (2010).


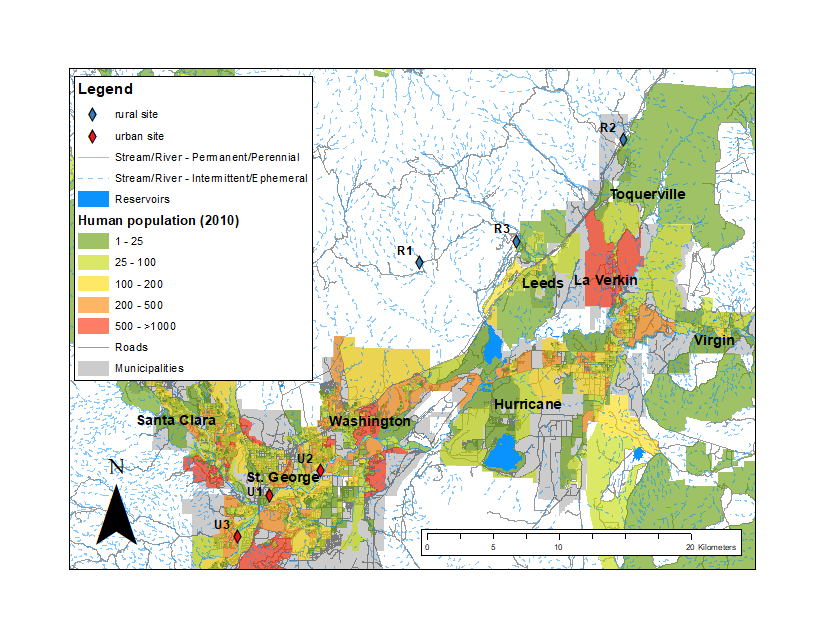


Figure S2: Site-specific invertebrate isotope ratios, showing that ants (pink) varied little between 2014 and 2017. Lizard means (± SD) are shown in black. Sample sizes given in Fig. S1 (invertebrates) and Table 1 (lizards). Mean (±SD) difference between ants from 2014 and 2017 was 1.8 ± 1.5, mean difference between ants from 2017 and other invertebrates from 2017 was 6.6 ± 2.8. Site U2 was completely developed in 2015 and could not be resampled in 2017.


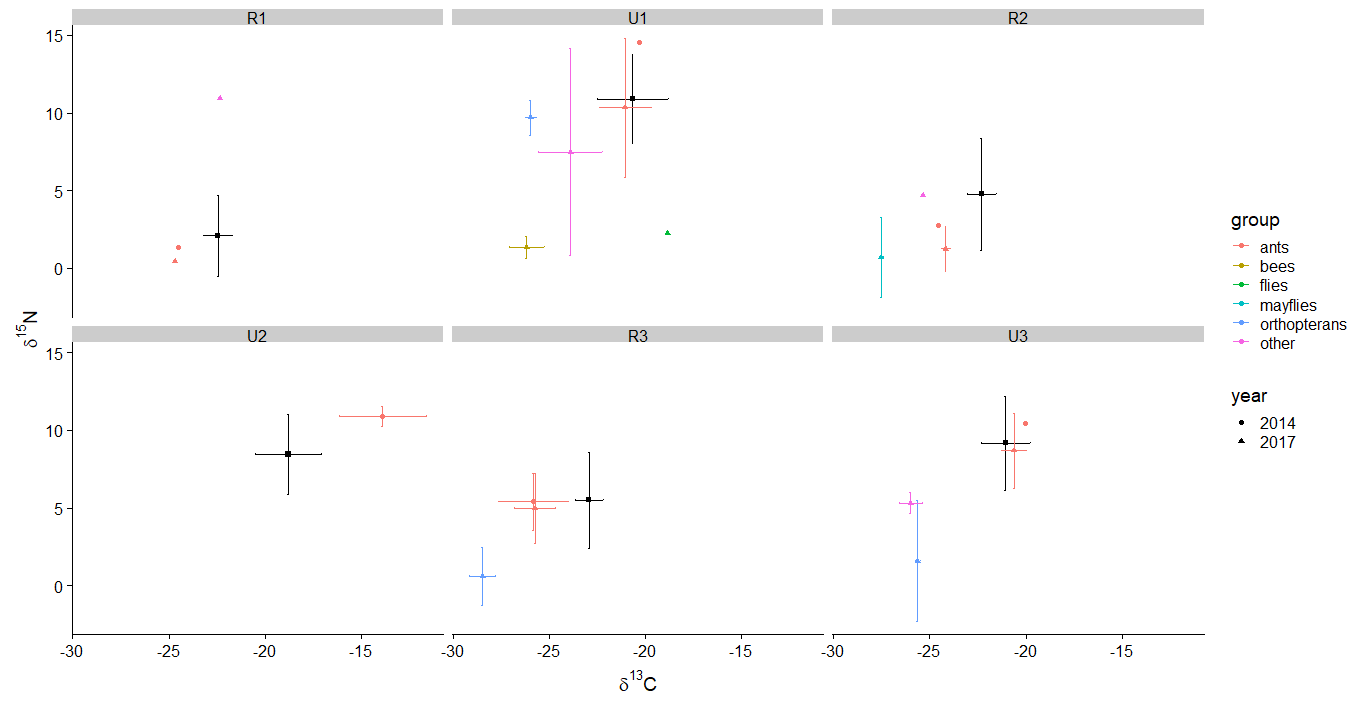


Table S1: Stable nitrogen and carbon isotope signatures of lizards, insects, and plants sampled at each site. U2 sampled 2013 & 2014 only.

| site | C_3_/C_4_/CAM | Taxon | N | $\bar{x}$ δ^15^N (± SD) | $\bar{x}$ δ^13^C (± SD) |
| --- | --- | --- | --- | --- | --- |
| R1 (BH) |  | Lizards | 103 | 2.1 ± 2.6 | -22.4 ± 0.8 |
|  |  | Ants | 2 | 0.9 ± 0.7 | -24.6 ± 0.2 |
|  | C_3_ | *Apocynum* | 3 | 53.8 ± 38.2 | -30.8 ± 1.4 |
|  |  | *Artemisia* | 1 | -1.2 | -25.7 |
|  |  | Brassicaceae | 3 | 27.5 ± 16.3 | -30.3 ± 0.7 |
|  |  | *Lupinus* | 3 | 3.5 ± 3.5 | -30.7 ± 0.1 |
|  |  | *Penstemon* | 4 | 17.5 ± 16.0 | -28.6 ± 1.7 |
|  |  | *Populus* | 1 | -2.4 | -28.7 |
|  |  | *Quercus* | 1 | -3.1 | -27.4 |
|  | C_3_/CAM | *Opuntia* | 3 | 84.6 ± 6.1 | -14.8 ± 1.7 |
|  | C_4_ | *Achnatherum* | 3 | 51.6 ± 26.0 | -25.0 ± 7.9 |
| U1 (BL) |  | Lizards | 96 | 10.9 ± 2.8 | -20.7 ± 1.8 |
|  | Invertebrates | Ants | 6 | 11.0 ± 4.4 | -20.9 ± 1.2 |
|  |  | Orthopterans | 2 | 9.7 ± 1.1 | -26.0 ± 0.3 |
|  |  | Dipterans | 1 | 2.3 | -18.9 |
|  |  | Bees | 2 | 1.4 ± 0.7 | -26.2 ± 0.9 |
|  | C_3_ | *Anisantha* | 1 | 12.4 | -23.5 |
|  |  | *Bromus* | 1 | 12.4 | -23.5 |
|  |  | *Cheilanthes* | 1 | 2.8 | -30.6 |
|  |  | *Chrysothamnus* | 3 | 23.1 ± 11.3 | -29.1 ± 0.5 |
|  |  | *Medicago* | 3 | .4 ± 9.4 | -28.8 ± 0.3 |
|  |  | *Poa* | 3 | 26.5 ± 23.4 | -26.6 ± 0.9 |
|  |  | *Tamarix* | 4 | 5.2 ± 3.8 | -27.6 ± 1.7 |
|  | C_4_ | *Achnatherum* | 1 | 9.5 | -16.1 |
|  |  | *Salsola* | 2 | 9.7 ± 2.0 | -13.8 ± 0.5 |
|  | CAM | *Salsola* | 2 | 9.7 ± 2.0 | -13.8 ± 0.5 |
| R2 (BR) |  | Lizards | 99 | 4.8 ± 3.6 | 22.3 ± 0.8 |
|  | Invertebrates | Ants | 4 | 1.6 ± 1.4 | -24.3 ± 0.3 |
|  |  | Ephemeropterans | 2 | 0.7 ± 2.6 | -27.5 ± 0.1 |
|  | C_3_ | *Dalea* | 3 | -4.9 ± 2.3 | -27.9 ± 1.0 |
|  |  | *Ephedra* | 1 | 2.5 | -26.7 |
|  |  | *Erigeron* | 3 | 1.8 ± 4.5 | -30.5 ± 1.4 |
|  |  | *Haplopappus* | 3 | 71.7 ± 49.4 | -29.2 ± 1.1 |
|  |  | *Salvia* | 3 | 1.7 ± 4.5 | -29.3 ± 0.3 |
|  | C_4_ | *Achnatherum* | 1 | 1.7 | -27.8 |
|  |  | *Aristida* | 2 | 4.8 ± 0.8 | -14.9 ± 0.1 |
| U2 (DI) |  | Lizards | 46 | 8.5 ± 2.6 | 18.8 ± 1.7 |
|  |  | Ants | 2 | 10.9 ± 0.66 | 13.9 ± 2.3 |
|  | C_3_ | *Anisantha* | 1 | 6.4 | -24.3 |
|  |  | *Bromus* | 1 | 6.4 | -24.3 |
|  |  | *Populus* | 1 | -0.9 | -27.8 |
|  |  | *Salix* | 1 | 2.3 | -26.8 |
|  | C_4_ | *Kochia* | 1 | 7.5 | -15.5 |
| R3 (LC) |  | Lizards | 98 | 5.5 ± 3.1 | 22.9 ± 0.7 |
|  | Invertebrates | Ants | 6 | 5.2 ± 1.8 | -25.8 ± 1.3 |
|  |  | Orthopterans | 3 | 0.6 ± 1.9 | -28.5 ± 0.7 |
|  | C_3_ | *Artemisia* | 4 | 15.7 ± 20.5 | -29.5 ± 1.3 |
|  |  | *Asteraceae* | 3 | 7.1 ± 20.3 | -29.9 ± 0.3 |
|  |  | *Bromus* | 3 | 28.2 ± 30.9 | -28.6 ± 1.3 |
|  |  | Fabaceae | 3 | -2.2 ± 1.4 | -27.3 ± 0.9 |
|  |  | *Penstemon* | 4 | 1.3 ± 2.8 | -30.0 ± 0.9 |
|  |  | *Populus* | 1 | -.3 | -29.3 |
|  |  | *Quercus* | 1 | 1.4 | -27.3 |
|  |  | *Rhus* | 3 | 4.5 ± 3.5 | -27.8 ± 0.5 |
|  |  | *Salix* | 1 | -.3 | -30.9 |
| U3 (MW) |  | Lizards | 108 | 9.2 ± 3.0 | -21.1 ± 1.3 |
|  | Invertebrates | Ants | 4 | 9.1 ± 2.2 | -20.5 ± 0.6 |
|  |  | Orthopterans | 2 | 1.6 ± 3.9 | -25.6 ± 0.1 |
|  | C_3_ | *Ambrosia* | 3 | 21.5 ± 19.2 | -32.7 ± 0.8 |
|  |  | *Bromus* | 2 | 5.9 ± 0.5 | -24.8 ± 0.1 |
|  |  | *Citrus* | 1 | 12.1 | -26.4 |
|  |  | *Ericameria* | 3 | 4.7 ± 5.7 | -27.0 ± 1.0 |
|  |  | *Populus* | 1 | 5.1 | -25.6 |
|  |  | *Salix* | 1 | 10.1 | -27.0 |
|  |  | *Tamarix* | 1 | 11.6 | -23.9 |
|  | C_4_ | *Atriplex* | 3 | 12.8 ± 9.3 | -17.6 ± 2.4 |
